# Supplementary material for: Confirmation of the local establishment of alien invasive turtle, Pseudemys peninsularis, in South Korea, using eggshell DNA
Source: PLoS One. 2023 Feb 16;18(2):e0281808. doi: 10.1371/journal.pone.0281808 (PMC9934327; doi:10.1371/journal.pone.0281808)

PLOS ONE

Confirmation of the local establishment of alien invasive turtle, Pseudemys peninsularis, in South Korea using eggshell DNA

--Manuscript Draft--

**Manuscript Number:**	PONE-D-22-32805
**Article Type:**	Research Article
**Full Title:**	Confirmation of the local establishment of alien invasive turtle, Pseudemys peninsularis, in South Korea using eggshell DNA
**Short Title:**	Eggshell DNA in identifying freshwater turtle species
**Corresponding Author:**	Ha-Cheol Sung<br>Chonnam National University<br>Gwangju, N/A KOREA, REPUBLIC OF
**Keywords:**	Invasive species; conservation; DNA technology; artificial hatching; egg characteristics
**Abstract:**	Alien invasive species are posing conservation challenges worldwide. Pet trade, one of the many ways, is worsening the situation. Especially, pet turtles have been released into nature due to their longer life span and peoples' religious and traditional beliefs. While the information on the successful local establishment and subsequent dispersal into new habitats is required to designate an invasive and ecosystem-disturbing species, alien freshwater turtle nests have always been hard to find and identify in nature. Because one should identify nests by the eggs, which do not always guide properly, as adults abandon the sites quickly. We thought the recent advancement in DNA technology may help improve the situation. We studied Pseudemys peninsularis, one of the most traded freshwater turtle pet species, which has already been reported from a wide range of wild areas in South Korea. Yet, it is not designated as ecosystem-disturbing species due to a lack of adequate information on their local reproduction and establishment. We conducted surveys and found two nests in Jeonpyeongje Neighborhood Park, Maewol-dong, Seo-gu, Gwangju. We developed the methodology for extracting DNA from the eggshells and successfully identified the nests by phylogenetic analysis and verified through egg characteristics and morphological features of artificially hatched juveniles. This was the first successful initiative to extract DNA from freshwater turtle eggshells. We believe it will help future researchers identify the alien invasive turtle nests and develop their control and management policies. In addition, our study also included comparative descriptions and schematic diagrams of the eggs of eight freshwater turtles, including a native and three ecosystem-disturbing species, from South Korea. We urged an immediate designation of P. peninsularis as an ecosystem-disturbing species considering its local establishment, distribution range, and potential negative impact on native ecosystems.
**Order of Authors:**	Ha-Cheol Sung
	Cheon Seung-Ju
	Md Mizanur Rahman, Ph.D.
	Ji-A Lee
	Seung-Min Park
	Jae-Hong Park
	Dong-Hyun Lee
**Additional Information:**	
**Question**	**Response**
**Financial Disclosure**<br><br>Enter a financial disclosure statement that describes the sources of funding for the	H-CS: RE201807039; Korea Environment Industry & Technology Institute.<br><br>The funders had no role in study design, data collection and analysis, decision to publish, or preparation of the manuscript.

Powered by Editorial Manager® and ProduXion Manager® from Aries Systems Corporation

work included in this submission. Review the submission guidelines for detailed requirements. View published research articles from *PLOS ONE* for specific examples.<br><br>This statement is required for submission and **will appear in the published article** if the submission is accepted. Please make sure it is accurate.<br><br>**Unfunded studies**<br>Enter: *The author(s) received no specific funding for this work.*<br><br>**Funded studies**<br>Enter a statement with the following details:<br>• Initials of the authors who received each award<br>• Grant numbers awarded to each author<br>• The full name of each funder<br>• URL of each funder website<br>• Did the sponsors or funders play any role in the study design, data collection and analysis, decision to publish, or preparation of the manuscript?<br>• **NO** - Include this sentence at the end of your statement: *The funders had no role in study design, data collection and analysis, decision to publish, or preparation of the manuscript.*<br>• **YES** - Specify the role(s) played.<br><br>* typeset	
**Competing Interests**<br><br>Use the instructions below to enter a competing interest statement for this submission. On behalf of all authors, disclose any competing interests that could be perceived to bias this work—acknowledging all financial support and any other relevant financial or non-financial competing interests.<br><br>This statement is required for submission and **will appear in the published article** if the submission is accepted. Please make sure it is accurate and that any funding sources listed in your Funding Information	The authors have declared that no competing interests exist.

later in the submission form are also declared in your Financial Disclosure statement.<br><br>View published research articles from *PLOS ONE* for specific examples.<br><br>**NO authors have competing interests**<br><br>Enter: *The authors have declared that no competing interests exist.*<br><br>**Authors with competing interests**<br><br>Enter competing interest details beginning with this statement:<br><br>*I have read the journal's policy and the authors of this manuscript have the following competing interests: [insert competing interests here]*<br><br>* typeset	
**Ethics Statement**<br><br>Enter an ethics statement for this submission. This statement is required if the study involved:<br><br>• Human participants<br>• Human specimens or tissue<br>• Vertebrate animals or cephalopods<br>• Vertebrate embryos or tissues<br>• Field research<br><br>Write "N/A" if the submission does not require an ethics statement.<br><br>General guidance is provided below. Consult the submission guidelines for detailed instructions. **Make sure that all information entered here is included in the Methods section of the manuscript.**	This study was approved by the Yeongsangang River Basin Environmental Office of the Korea Ministry of Environment (permission number: 2021-8). The experiments were conducted following the instructions and ethical permissions from the ethical committee, Laboratory Animal Research Center, Chonnam National University, South Korea (Certificate No. 2020-154).

**Format for specific study types**<br><br>**Human Subject Research (involving human participants and/or tissue)**<br>• Give the name of the institutional review board or ethics committee that approved the study<br>• Include the approval number and/or a statement indicating approval of this research<br>• Indicate the form of consent obtained (written/oral) or the reason that consent was not obtained (e.g. the data were analyzed anonymously)<br><br>**Animal Research (involving vertebrate animals, embryos or tissues)**<br>• Provide the name of the Institutional Animal Care and Use Committee (IACUC) or other relevant ethics board that reviewed the study protocol, and indicate whether they approved this research or granted a formal waiver of ethical approval<br>• Include an approval number if one was obtained<br>• If the study involved *non-human primates*, add *additional details* about animal welfare and steps taken to ameliorate suffering<br>• If anesthesia, euthanasia, or any kind of animal sacrifice is part of the study, include briefly which substances and/or methods were applied<br><br>**Field Research**<br><br>Include the following details if this study involves the collection of plant, animal, or other materials from a natural setting:<br>• Field permit number<br>• Name of the institution or relevant body that granted permission	
**Data Availability**<br><br>Authors are required to make all data underlying the findings described fully available, without restriction, and from the time of publication. PLOS allows rare exceptions to address legal and ethical concerns. See the PLOS Data Policy and FAQ for detailed information.	Yes - all data are fully available without restriction

Powered by Editorial Manager® and ProduXion Manager® from Aries Systems Corporation

A Data Availability Statement describing where the data can be found is required at submission. Your answers to this question constitute the Data Availability Statement and **will be published in the article**, if accepted.<br><br>**Important:** Stating 'data available on request from the author' is not sufficient. If your data are only available upon request, select 'No' for the first question and explain your exceptional situation in the text box.<br><br>Do the authors confirm that all data underlying the findings described in their manuscript are fully available without restriction?	
**Describe where the data may be found in full sentences. If you are copying our sample text, replace any instances of XXX with the appropriate details.**<br><br>• If the data are **held or will be held in a public repository**, include URLs, accession numbers or DOIs. If this information will only be available after acceptance, indicate this by ticking the box below. For example: *All XXX files are available from the XXX database (accession number(s) XXX, XXX.*).<br>• If the data are all contained **within the manuscript and/or Supporting Information files**, enter the following: *All relevant data are within the manuscript and its Supporting Information files.*<br>• If neither of these applies but you are able to provide **details of access elsewhere**, with or without limitations, please do so. For example:<br><br>*Data cannot be shared publicly because of [XXX]. Data are available from the XXX Institutional Data Access / Ethics Committee (contact via XXX) for researchers who meet the criteria for access to confidential data.*<br><br>*The data underlying the results presented in the study are available from (include the name of the third party*	All relevant data are within the manuscript and its Supporting Information files.

and contact information or URL).<br>• This text is appropriate if the data are owned by a third party and authors do not have permission to share the data.<br><br>* typeset	
Additional data availability information:	

Powered by Editorial Manager® and ProduXion Manager® from Aries Systems Corporation

Manuscript

Click here to access/download;Manuscript;Manuscript_MMR.docx

Title: Confirmation of the local establishment of alien invasive turtle, *Pseudemys peninsularis*, in South Korea using eggshell DNA

Short title: Eggshell DNA in identifying freshwater turtle species

Authors and affiliations: Cheon Seung-Ju[1,#], Md Mizanur Rahman[2,#], Ji-A Lee [1], Seung-Min Park [1], Jae-Hong Park [1], Dong-Hyun Lee[2], Ha-Cheol Sung [2,*]

[1] Department of Biological Sciences and Biotechnology, Chonnam National University, 61186 Gwangju, South Korea

[2]Department of Biological Sciences, Chonnam National University, 61186 Gwangju, South Korea

[#]These authors contributed equally

[*] Corresponding author: shcol2002@jnu.ac.kr

Abstract

Alien invasive species are posing conservation challenges worldwide. Pet trade, one of the many ways, is worsening the situation. Especially, pet turtles have been released into nature due to their longer life span and peoples' religious and traditional beliefs. While the information on the successful local establishment and subsequent dispersal into new habitats is required to designate an invasive and ecosystem-disturbing species, alien freshwater turtle nests have always been hard to find and identify in nature. Because one should identify nests by the eggs, which do not always guide properly, as adults abandon the sites quickly. We thought the recent advancement in DNA technology may help improve the situation. We studied *Pseudemys peninsularis*, one of the most traded freshwater turtle pet species, which has already been reported from a wide range of wild areas in South Korea. Yet, it is not designated as ecosystem-disturbing species due to a lack of adequate information on their local reproduction and establishment. We conducted surveys and found two nests in Jeonpyeongje Neighborhood Park, Maewol-dong, Seo-gu, Gwangju. We developed the methodology for extracting DNA from the eggshells and successfully identified the nests by phylogenetic analysis and verified through egg characteristics and morphological features of artificially hatched juveniles. This was the first successful initiative to extract DNA from freshwater turtle eggshells. We believe it will help future researchers identify the alien invasive turtle nests and develop their control and management policies. In addition, our study also included comparative descriptions and schematic diagrams of the eggs of eight freshwater turtles, including a native and three ecosystem-disturbing species, from South Korea. We urged an immediate designation of *P. peninsularis* as an ecosystem-disturbing species considering its local establishment, distribution range, and potential negative impact on native ecosystems.

Introduction

In the era of conservation challenges, invasive species is a growing global concern [1,2]. Among many other ways, the pet trade is accelerating the spread of invasive species around the world [3]. The countries like South Korea, which are already well-known as pet trade epicenters, are at high risk of getting invasive species into their nature [4]. Although at least 677 herpetofauna are reported from pet shops in Korea, species from Testudines have been more frequently recorded from the wild [5]. The report of alien turtle species in nature mainly resulted from the abandonment by the pet owners and release during religious events [6,7]. More than 13 alien turtle species are currently found in the wild [8,9]. To date, to facilitate the

management and have legal ground, *Trachemys* spp., *Pseudemys concinna*, *Mauremys sinensis*, *Macrochelys temminckii*, *Pseudemys nelsoni*, and *Chelydra serpentina*, among the wild reported alien turtles, have been designated as ecosystem-disturbing species [10].

However, it is necessary to ensure the successful local establishment and subsequent dispersal and secondary spread into new habitats after the introduction of a species to designate it as an invasive and ecosystem-disturbing species [11]. In the case of reptiles, a species is considered to be established if there is evidence of maintaining a population by laying eggs in the local environment after getting released, abandoned, or escaped [12,13]. Unfortunately, we have little information on the nest sites and eggs of most of the alien turtle species, including *Pseudemys peninsularis*, recorded from the wild of South Korea.

Freshwater turtle nests have always been hard to find in nature [14]. Furthermore, the identification of turtle nests is largely depended on the identification of the nest-building and egg-laying individuals [15], while some species take a very little amount of time for it [16]. Thus, few researchers suggested using egg external features for turtle nest identification [17,18], whereas others argued it was ineffective due to overlapping egg diameters [19]. Like many other animal groups, recent studies showed the successful use of DNA barcoding in identifying herpetofauna [20–22]. Hence, we hypothesized that eggshells can be used as a source of DNA and in identifying freshwater turtle nests and species, including *P. peninsularis*, which is already well-practiced in birds and sea turtles [23–25].

We studied *P. peninsularis*, one of the most traded freshwater turtle pet species in South Korea which has already been reported from a wide range of wild areas [26]. Yet, it is not designated as ecosystem-disturbing species because of a lack of adequate information on the successful reproduction and secondary spread in the local environment. Considering the absence of information on the nests and eggs in nature, confirmation is needed for the establishment of this species in the native ecosystems. Thus, we conducted surveys to discover *P. peninsularis* nests and eggs in the wild of South Korea. We developed a method for extracting genomic DNA from freshwater turtle eggshells and used partial mitochondrial DNA sequence for molecular identification of the species. This is the first initiative for extracting DNA from freshwater turtle eggshells and using it in species identification. Thus, we measured the egg characteristics and checked the morphology of the hatchlings after hatched the eggs artificially to verify the molecular-based identification and validate the sequences we amplified from the eggshells. Furthermore, we presented the comparison among egg characteristics and

external features of Korea inhabiting eight turtle species, including a native and three ecosystem-disturbing species. Finally, we suggested designating *P. peninsularis* as an ecosystem-disturbing species in Korea and strengthening its control and management policies.

Material and Method

Discovery of nests and egg collection

Considering previous reports of many alien turtles, the Jeonpyeongje Neighborhood Park, Maewol-dong, Seo-gu, Gwangju (35° 6'55.14"N 126°50'54.24"E; Fig 1), was surveyed for the observation of possible nest sites of *P. peninsularis*. The Jeonpyeongje Neighborhood Park, has an area of 4.699 ha. It has a reservoir, which is easily accessible by a wooden bridge and is reported to contain many alien turtles, including *T. scripta elegans*, *T. scripta troostii*, *P. concinna*, and *P. peninsularis*. Thus, we surveyed the reservoir and discovered two nests of *P. peninsularis*. We recorded the external features and measured the eggs for comparing and identifying the species. The damaged eggs were excluded from the measurements. The eggs were collected for further analysis.

Fig 1. Distribution of *Pseudemys peninsularis* and the study area. (A) Distribution of *P. peninsularis* across the world. The red star indicates the location of South Korea. (B) Jeonpyeongje Neighborhood Park with the nest sites indicated by the yellow stars. (C) A nest with *P. peninsularis* eggs. (D) Map of South Korea with study site at Gwangju indicated by a green star.

Identification and comparison of eggs

To confirm the identity of the species and the nest, we measured and compared the egg parameters with previous literature. We followed Congdon and Gibbons (1985) and Iverson and Ewert (1991) [17,27]. We recorded the clutch size of the nests, shell type, mass, length, width, and elongation of the eggs.

DNA extraction

We used DNeasy Blood & Tissue kit (QIAGEN, Germany) and followed a partially modified manufacturer’s protocol for DNA extraction. First, we took 60 mg of egg membrane from the

eggshell and mixed it with ATL buffer 200µl, proteinase K 20µl, and dithiothreitol (DTT; 1mg/ml) 9.8µl to react 24h at 55°C. After centrifugation at 18,000xg for a minute, the supernatant was transferred to a new 1.5 ml tube and mixed with AL buffer 200µl and ethanol (95-100%) 200µl. Then the samples were transferred to the Qiagen column and centrifuged again at 6,000xg for a minute. After that, we added AW1 buffer 500µl to the column and again centrifuged at 6,000xg for a minute. In the next step, we added AW2 buffer 500µl to the column and centrifuged at 20,000xg for 3 minutes. After the column was transferred to a new 1.5ml tube and eluted in 70µl and centrifuged at 6,000xg for a minute. Polymerase chain reaction (PCR) amplifications were performed using Solg Pfu DNA polymerase (Solgent, Korea) to confirm the mitochondrial DNA sequence. We amplified a Control region, a widely used species-specific sequence, using DES_1 (5'-GCATTCATCTATTTTCCGTTAGCA -3')/DES_2 (5'-GGATTTAGGGGTTTGACGAG -3') [28].

Phylogenetic analysis

To confirm the identity of the specimens via DNA barcoding and to evaluate their matrilineal relationships, we analyzed our new sequences together with relevant homologous sequences in GenBank (Table 1). Based on previous phylogenetic analyses [29] we used *Chrysemys picta* as the outgroup. All sequences were aligned by MEGA X [30]. We inferred matrilineal genealogy using Bayesian Inference (BI) analyses and Maximum Likelihood (ML) methods. We used MrBayes 3.1.2 [31] for BI analyses, while the best model was assessed with the software JMODELTEST [32]. The GTR+Gamma was found to be the best model. The analyses were conducted with 10 million generations and sampled every 1000 generations. The convergence was judged in the software TRACER version 1.6 [33] using the average standard deviation of split frequencies (< 0.01) and the ESS values (> 200). We used four Markov Chain Monte Carlo chains to perform two independent runs and discarded 25% of the tree as burn-in. Finally, the obtained tree was visualized with the help of FigTree 1.4.2 [34]. The ML tree was constructed through RAxML 7.0.4 [35], with the GTR substitution model, gamma-distributed rate heterogeneity among sites, and the invariable proportion sites estimated from the data. The analysis used 1000 bootstrap pseudo-replicates. We considered the nodes with equal or more than 0.95 Bayesian Posterior Probabilities (BPP) in the BI tree and equal or more than 70% Bootstrap Support (BS) in the ML tree as well supported. Furthermore, genetic distances were calculated under the uncorrected p-distance model using MEGA X [30].

Table 1. List of sequences used in the present study. The bold fonts indicate the *de novo* sequence obtained from the *P. peninsularis* eggshells in the present study.

Serial No.	Description	voucher	region	Accession number	Locality	Reference
1	*P. peninsularis*		D-loop	***	South Korea	Present study
2	*P. peninsularis*		D-loop	***	South Korea	Present study
3	*P. peninsularis*		D-loop	NC063096	South Korea	[36]
4	*P. peninsularis*	FLMNH10952	D-loop	GQ395743	USA	[37]
5	*P. peninsularis*	FLMNH150993	D-loop	GQ395739	USA	[37]
6	*P. peninsularis*	HBS108722	D-loop	KC687239	USA	[29]
7	*P. peninsularis*	FTA821	D-loop	KC687237	USA	[29]
8	*P. peninsularis*	FLMNH151505	D-loop	KC687236	USA	[29]
9	*P. peninsularis*	CRI3953	D-loop	KC687235	USA	[29]
10	*P. concinna*	HBS123561	D-loop	KC687215	USA	[29]
11	*P. concinna*	HBS123555	D-loop	KC687213	USA	[29]
12	*P. concinna*	HBS117819	D-loop	KC687212	USA	[29]
13	*P. nelsoni*	HBS108599	D-loop	KC687233	USA	[29]
14	*P. nelsoni*	FTA832	D-loop	KC687232	USA	[29]
15	*P. nelsoni*	MVZ 164993	D-loop	MT424571	USA	Unpublished
16	*P. alabamensis*	HBS123564	D-loop	KC687171	USA	[29]
17	*P. alabamensis*	HBS123559	D-loop	KC687170	USA	[29]
18	*P. alabamensis*	HBS123557	D-loop	KC687169	USA	[29]
19	*P. rubriventris*	USNM544373	D-loop	KC687243	USA	[29]
20	*P. rubriventris*	MCZ188655	D-loop	KC687242	USA	[29]
21	*P. rubriventris*	MCZ188654	D-loop	KC687241	USA	[29]
22	*P. texana*	RCT2	D-loop	KX558427	USA	[29]
23	*P. texana*	RCT66	D-loop	KC687247	USA	[29]
24	*P. texana*	RCT2	D-loop	KC687245	USA	[29]
25	*Chrysemys picta*		D-loop	JN993967	USA	Unpublish

Artificial incubation and identification of the hatchlings

The collected eggs were incubated at 28 – 30℃. This temperature range is proven to give a high success rate in a short incubation period [38]. The hydrated moss was placed underneath, which was replaced every two weeks, to maintain the humidity. The eggs were hatched in 47 – 50 days. The hatchlings were identified through external morphological features.

Ethical permit

This study was approved by the Yeongsangang River Basin Environmental Office of the Korea Ministry of Environment (permission number: 2021-8). The experiments were conducted following the instructions and ethical permissions from the ethical committee, Laboratory Animal Research Center, Chonnam National University, South Korea (Certificate No. 2020-154).

Result

Phylogenetic analysis

The sequences from our study clustered with other sequences of *P. peninsularis* from the GenBank in the phylogenetic tree. We found *P. peninsularis* to be a sister clade to *P. nelsoni*. Whereas, *P. rubiventris* was a sister clade to *P. peninsularis*, *P. nelsoni*, *P. concinna*, *P. albamensis*, and *P. texana*. However, the taxa of the latter clade were highly inclusive with strong node supports (Fig 2A). Furthermore, the BLAST results also revealed similar patterns to phylogenetic analysis. BLAST in NCBI showed the generated sequences had a 99.80% similarity with the sequences of *P. peninsularis* deposited in the GenBank. The *de novo* sequences showed very low genetic distances with the sequences of the same species from the GenBank than other species (Fig 2B). Our sequences had 0% to 1% uncorrected *p*-distances with other sequences of *P. peninsularis*.

Fig 2. Identification of the samples. (A) Phylogenetic tree based on mitochondrial DNA control region. Nodal support values placed before '/' are Bayesian Posterior Probabilities (BPP), and values placed after '/' are Maximum Likelihood Bootstraps (BS). In our tree '*' denotes greater or equals to 0.95 BPP and 70% BS, '#' denotes 0.85–0.94 BPP and 60–69% BS, and '–' denotes below 0.85 BPP and 60% BS. The numbers in the parentheses correspond to the serial number of Table 1. The bold fonts indicate the *de novo* sequences. (B) Pairwise genetic distance (*p*-

distance). We excluded the outgroup from the calculation. The numbers correspond to the serial number of Table 1. (C) Dorso-lateral view of a hatchling of *P. peninsularis*. (D) Ventral view of a hatchling of P. peninsularis.

Nest and egg characteristics

The clutch size of the two nests was 27 and 07 respectively. As 10 eggs were damaged during the collection, we measured 24 eggs for the identifications. The eggs were oval-elongated shaped and white in color. The eggshells were of parchment type (Fig. 1C). We found a length of 31.43mm (±0.878), a width of 22.62mm (±0.549), an elongation of 1.39 (±0.067), a mass of 9.15g (±0.607). In addition, the compilation of egg properties of eight freshwater turtle species from South Korea revealed differences in species level except for *Trachemy scripta* and *Graptemys ouachitensis* (Fig 3). These two species had the same egg elongation of 1.64, while others had different egg elongations and thus different shapes (Table 2).

Fig 3. Egg shapes and eggshells of freshwater turtles found in South Korea. (A) *Pseudemys* *peninsularis*; (B) *P. concinna*; (C) *Trachemys scripta*; (D) *Graptemys ouachitensis*; (E) *Graptemys geographica*; (F) *Chrysemys picta*; (G) *Chelydra serpentina*; (H) *Mauremys reevesii*. The eggs without outlines indicate parchment shells and the egg with a black outline indicates a brittle shell.

Table 2. Egg characteristics of eight freshwater turtles from South Korea. A = *Pseudemys peninsularis*; B = *Pseudemys concinna*; C = *Trachemys scripta*; D = *Graptemys ouachitensis*; E = *Graptemys geographica*; F = *Chrysemys picta*; G = *Chelydra serpentina*; H = *Mauremys reevesii*; α = Parchment; β = Brittle. * = does not have the egg length in the corresponding literature and is thus excluded from the calculation. i = Present study; ii = Congdon and Gibbons (1985) [17]; iii = Iverson and Ewert (1991) [27].

Species		A	B	C	D	E	F	G	H
Shell type		α	α	α	α	α	α	α	β
Mass (g)	i	9.15							

	ii		15.44	9.74	10.06	10.57	6.67	9.91	10.17
	iii	11.49	11.99	10.52			6.17	9.63	
	Mean	10.32	13.72	10.13	10.06	10.57	6.42	9.77	10.17
Length (mm)	i	31.43							
	ii		42.03	35.63	36.18	37.20	31.56	26.49	42.88
	iii	36.22	36.36	36.39			33.22		
	Mean	33.83	39.20	36.01	36.18	37.20	32.39	26.49	42.88
Width (mm)	i	22.62							
	ii		24.92	21.83	22.08	22.04	19.10	25.25	19.47
	iii	23.15	23.90	22.17			17.28	25.78*	
	Mean	22.89	24.41	22.00	22.08	22.04	18.19	25.25	19.47
Elongation		1.48	1.61	1.64	1.64	1.69	1.78	1.05	2.20

Hatchling characteristics

The carapace of hatchlings was dark green with parallel yellow stripes. The marginal shells were with a marginal yellow line. There was a green-colored mid-dorsal keel. The dorsal side legs, tail, and neck were mostly dark green with longitudinal yellow stripes. The yellow stripes on the head form a U-shape behind the eyes. The plastron was unhinged and mostly yellow in color (Fig 2C,D).

Discussion

We found two *P. peninsularis* turtle nests in Jeonpyeongje Neighborhood Park, Maewol-dong, Seo-gu, Gwangju, South Korea (Fig 1B,C,D), and successfully identified them by combining the egg characteristics, morphological features of the hatchlings, and phylogenetic analysis of partial mitochondrial DNA from the eggshells. This was the first initiative to extract DNA from eggshells, which is already well-practiced in sea turtles [24,25]. Although our initiative was successful, extracting DNA from eggshells is challenging as it is expected to get a lower concentration than that may yield from muscle or blood [39]. The low concentration and poor template quality may lead to amplification failure [40,41]. However, using DNA from eggshells is convenient, especially, to identify the nests because it does not require the individual turtles [25,42]. Furthermore, freshwater turtle nests are difficult to find and are also difficult and time-

consuming to hatch the eggs to identify the nests [14]. Thus, although *P. peninsularis* is widely distributed in South Korea and in many other non-native countries, there are no reports on nests of this species in nature [26]. This is the first report of *P. peninsularis* nests in nature outside its native range [14].

However, the BLAST results and phylogenetic analysis of the partial mitochondrial DNA from our samples revealed a close relationship and low genetic distances between *P. peninsularis* and *P. nelsoni*, *P. concinna*, and *P. albamensis* (Fig 2B). This finding is similar to the previous studies [29,37]. The BLAST results suggested a high similarity between the novel sequences of *P. penisularis* and *P. nelsoni* (99.59%), *P. concinna* (99.39%), and *P. alabamensis* (98.99%). Similarly, our sequences showed low genetic distances with these species. The uncorrected *p*-distances between our sequences and *P. nelsoni*, *P. concinna*, *P. alabamensis*, *P. texana*, and *P. rubiventris* were 0.2 – 0.4%, 0.8% - 1.2%, 0.7 – 1.0%, 0.8% – 1.2%, and 1.6% – 2.2% respectively. The phylogenetic tree topology also supported the BLAST results and uncorrected *p*-distances by forming inclusive clades with strong node supports (Fig 2A). Although the clades of *P. peninsularis*, *P. nelsoni*, *P. concinna*, and *P. alabamensis* were inclusive and only *P. rubiventris* was a sister clade to them, our results recovered almost similar topology as previous genetic studies on the *Pseudemys* genus and supported the hypothesis of taxonomic confusions in this genus [29,37].

In addition to molecular and morphology-based species identifications, egg properties also can be used in turtle nest identification. Especially, egg elongation is useful in identifying turtle species [17,18]. We got an egg elongation of 1.48 for P. penisularis in the present study, which is similar to the previous studies [27]. Additionally, the compilation of data on egg properties of eight turtle species from South Korea revealed an egg elongation of 1.61, 1.69, 1.78, 1.05, and 2.20 for *P. concinna*, *Graptemys geographica*, *Chrysemys picta*, and *Mauremis reevesii* respectively (Table 2). The other two species, *Trachemys scripta* and *G. ouachitensis* had the same egg elongation, 1.64. Furthermore, we recovered the schematic diagram of the egg shapes based on the present and previous studies (Fig 3) [17,27], which showed the effectiveness of using egg properties in identifying turtle nests.

The identification and discovery of the freshwater turtle nests have always been very difficult for researchers [14]. Thus, the nests of *P. peninsularis* have not been reported from outside of its native range even after being widely used as a pet species and having a wide distribution in America, Europe, and Asia (Fig 1A) [43–47]. In South Korea, this species has

been reported from all over the country including Jeju island [26]. Considering the large body size and capability of basking in a high temperature (Ernst and Lovich, 2009; Munscher et al., 2015), this species has the potential to pose competition pressure and threats to the native species. In addition to proven negative impacts on other turtle species [50–52], this species has been reported to bear parasites like *Aeromonas enteropelogenes*, *A. hydrophila*, and *A. veronii*, potential to affect amphibian and human health [53]. Still, this species has not been designated as an ecosystem-disturbing species as it lacks confirmed information on its successful establishment and reproduction [11], which can be evident by reports on its nests in the local environment [12,13].

The present study has confirmed the successful establishment and reproduction of *P. peninsularis* in South Korea by identifying its nests in the local environment through traditional and molecular approaches. Thus, considering the distribution and potential negative impact of this alien species on native ecosystems and human beings, we urge immediate designation of *P. peninsularis* as an ecosystem-disturbing species and its control and management policies. In addition, our study is highly significant as it included comparative descriptions and schematic diagrams of the eggs of eight freshwater turtles from South Korea. It will help scientists in studying freshwater turtles, including invasive species, in this country. Furthermore, we have presented the first-ever successful use of eggshell DNA in identifying freshwater turtles. We believe it could be significant tool for future researches in identifying the alien invasive freshwater turtle nests and developing their control and management policies across the world.

Acknowledgements

This work was supported by Korea Environment Industry & Technology Institute (KEITI) through the Project for the Development of Biological Diversity Threats Outbreak Management Technology (RE201807039), funded by the Korea Ministry of Environment (MOE).

Competing interests

The authors declare no competing interests.

Author contributions

Seung-Ju Cheon: contributed to the sample collection, methodology, lab work, visualization, and writing- review, and editing. **Md mizanur Rahman:** contributed to the conceptualization, formal analysis, software, visualization, writing- original draft, review, editing, and critical feedback. **Ji-A Lee, Seung-Min Park, and Jae-Hong Park:** contributed to the field survey and review the draft. **Dong-Hyun Lee:** contributed to the conceptualization, supervision, and review of the draft. **Ha-Cheol Sung:** contributed to the conceptualization, supervision, funding acquisition, methodology, review, and critical feedback. All authors read and approved the final version of the manuscript.

Reference

1. Pimentel D. Economic and environmental impacts of invasive species and their management. Pesticides and you. 2001; 21:10–11.

2. Beck KG, Zimmerman K, Schardt JD, Jeffrey S, Ronald RL, Sarah R, et al. Invasive species defined in a policy context: Recommendations from the Federal Invasive Species Advisory Committee. Invasive Plant Science and Management. 2008; 1:414–421. https://doi.org/10.1614/IPSM-08-089.1.

3. Park SM, Rahman MM, Ham CH, Sung HC. The first record of an invasive reptile species, *Pelomedusa* cf. olivacea (Schweigger, 1812) (*Pelomedusidae*, Testudines), in the wild of South Korea. Check List. 2022; 18(5):989–993. https://doi.org/10.15560/18.5.989.

4. Park HR, Rahman MM, Park SM, Choi JH, Kang HJ, Sung HC. Risk assessment for the native anurans from an alien invasive species, American bullfrogs (*Lithobates catesbeianus*), in South Korea. Scientific Reports. 2022; 12(1):1–11.

https://doi.org/10.1038/s41598-022-17226-8.

5. Koo KS, Park HR, Choi JH, Sung HC. Present status of non-native amphibians and reptiles traded in Korean online pet shop. Korean Journal of Environment and Ecology. 2020; 34:106-114. https://doi.org/10.13047/KJEE.2020.34.2.106.

6. Agoramoorthy G, Hsu MJ. Religious freeing of wildlife promotes alien species invasion. BioScience. 2005; 55:5–6. https://doi.org/10.1641/0006-3568(2005)055[0006:RFOWPA]2.0.CO;2.

7. Liu X, McGarrity ME, Li Y. The influence of traditional Buddhist wildlife release on biological invasions. Conservation Letters. 2012; 5:107–114. https://doi.org/10.1111/j.1755-263X.2011.00215.x.

8. National Institute of Environment, NIE. Ecological studies of alien species (III). Ministry of Environment. 2016; pp 43-50.

9. Koo KS, Song S, Choi JH, Sung HC. Current distribution and status of non-native freshwater turtles in the wild, Republic of Korea. Sustainability. 2020; 12:4042. https://doi.org/10.3390/su12104042.

10. Ministry of Environment, MoE. Act on the conservation and use of biological diversity. 2022 [Act No. 2022-209, 28, Oct, 2022., Partial Amendment]. Notice of Designated Ecosystem Disturbance Organisms (law.go.kr).

11. Sakai AK, Allendorf FW, Holt JS, Lodge DM, Molofsky J, With KA, et al. The population biology of invasive species. Annual review of ecology and systematics. 2001; 32:305–332. https://doi.org/10.1146/annurev.ecolsys.32.081501.114037.

12. Shea K, Chesson P. Community ecology theory as a framework for biological invasions. Trends in Ecology & Evolution. 2002; 17:170–176. https://doi.org/10.1016/S0169-5347(02)02495-3.

13. Van Wilgen NJ, Wilson JRU, Elith J, Wintle BA, Richardson DM. Alien invaders and reptile traders: what drives the live animal trade in South Africa? Animal Conservation. 2010; 13:24–32. https://doi.org/10.1111/j.1469-1795.2009.00298.x

14. Zuffi M. Conservation biology of the European pond turtle *Emys orbicularis* (L.) in Italy.

Stapfia 2000; 69(149):219–228.

15. Schroeder BA, Foley AM, Bagley DA. Nesting patterns, reproductive migrations, and adult foraging areas of loggerhead turtles. In: Loggerhead Sea Turtles (eds Bolten AB & Witherington BE). 2003; pp. 114–124. Smithsonian, Washington, D.C.

16. Hirth HF. Some aspects of the nesting behavior and reproductive biology of sea turtles. Integrative and comparative biology. 1980; 20:507–523.

17. Iverson JB, Ewert MA. Physical characteristics of reptilian eggs and a comparison with avian eggs. Egg Incubation: Its Effect on Embryonic Development in Birds and Reptiles. Cambridge University Press, New York, New York, USA. 1991; 87–100.

18. Rose FL, Simpson TR, Manning RW. Measured and predicted egg volume of *Pseudemys texana* with comments on turtle egg shape. Journal of Herpetology. 1996; 30(3):433–435. https://doi.org/10.2307/1565188.

19. Miller JD. Reproduction in sea turtles. In: The Biology of Sea Turtles (eds Lutz PL & Musick JA). 1997; pp. 51–80. CRC Press, Boca Raton, Florida.

20. Nagy ZT, Sonet G, Glaw F, Vences M. First large-scale DNA barcoding assessment of reptiles in the biodiversity hotspot of Madagascar, based on newly designed COI primers. PLoS ONE. 2012; 7(3):e34506. doi:10.1371/journal.pone.0034506.

21. Rahman MM, Chen JM, Wu, YH Chen HM, Lwin YH., Murphy RW, et al. New country records for three species of frog from Myanmar including two genera (*Nasutixalus* and *Oreolalax*). Zootaxa. 2020; 4742(3):531–542. https://doi.org/10.11646/zootaxa.4742.3.7.

22. Rahman MM, Nneji LM, Adeniyi AC, Chen JM, Eniang EA, Oladipo SO, et al. Amphibian assemblages and diversity patterns in two forest ecosystems of Southern Nigeria. African Journal of Ecology. 2020; 58:815–827. https://doi.org/10.1111/aje.12776.

23. Tsai WLE, Schedl ME, Maley JM, McCormack JE. More than skin and bones: Comparing extraction methods and alternative sources of DNA from avian museum specimens. Molecular Ecology Resources. 2019; 20(5):1220–1227. https://doi.org/10.1111/1755-0998.13077.

24. Moore MK, Bemiss JA, Rice SM, Quattro JM, Woodley CM. Use of restriction length polymorphisms to identify sea turtle eggs and cooked meats to species. Conservation

Genetics. 2003; 4:95–103. https://doi.org/10.1023/A:1021881319271.

25. Shamblin BM, Dodd MG, Williams KL, Frick MG, Bell R, Nairn CJ. Loggerhead turtle eggshells as a source of maternal nuclear genomic DNA for population genetic studies. Molecular Ecology Resources. 2011; 11:110–115. https://doi.org/10.1111/j.1755-0998.2010.02910.x.

26. National Institute of Environment, NIE. Nationwide survey of nonnative species in Korea (V). Ministry of Environment. 2019; 144 pp.

27. Congdon JD, Gibbons JW. Egg components and reproductive characteristics of turtles: Relationships to body size. Herpetologica. 1985; 41(2):194–205. http://www.jstor.org/stable/3892258.

28. Starkey DE, Shaffer HB, Burke RL, Forstner MR, Iverson JB, Janzen FJ, et al. Molecular systematics, phylogeography, and the effects of Pleistocene glaciation in the painted turtle (*Chrysemys picta*) complex. Evolution. 2003; 57:119–128. https://doi.org/10.1111/j.0014-3820.2003.tb00220.x.

29. Spinks PQ, Thomson RC, Pauly GB, Newman CE, Mount G, Shaffer HB. Misleading phylogenetic inferences based on single-exemplar sampling in the turtle genus *Pseudemys*. Molecular Phylogenetics and Evolution. 2013; 68:269–281. https://doi.org/10.1016/j.ympev.2013.03.031.

30. Kumar S., Stecher G., Li M., Knyaz C., and Tamura K. MEGA X: Molecular Evolutionary Genetics Analysis across computing platforms. Molecular Biology and Evolution. 2018; 35:1547–1549. https://doi.org/10.1093/molbev/msy096.

31. Ronquist F, Huelsenbeck JP. MrBayes 3: Bayesian phylogenetic inference under mixed models. Bioinformatics. 2003; 19(12):1572–1574. https://doi.org/10.1093/bioinformatics/btg180.

32. Posada D. JModelTest: phylogenetic model averaging. Molecular Biology and Evolution. 2008; 25:1253–1256. https://doi.org/10.1093/molbev/msn083.

33. Rambaut A, Drummond AJ, Suchard M. Tracer v1.6.0. 2013. Retrieved from http://beast.bio.ed.ac.uk/.

34. Rambaut A. Figtree v 1.4.0. 2012. http://tree.bio.ed.ac.uk/software/figtree/.

35. Stamatakis A. RAxML–VI–HPC: maximum likelihood–based phylogenetic analyses with thousands of taxa and mixed models. Bioinformatics. 2006; 22:2688–2690. https://doi.org/10.1093/bioinformatics/btl446.

36. Chung D, Park J, Cheon S, Park SM, Sung HC, Lee D H. Complete mitochondrial genome of the peninsula cooter (*Pseudemys peninsularis*, Testudines: Emydidae) in Korea. Mitochondrial DNA Part B. 2022; 7(8):1441–1442. https://doi.org/10.1080/23802359.2022.2107463.

37. Thomas GJ, Nelson DH, Morris AB. Phylogenetic relationships in the North American genus *Pseudemys* (Emydidae) inferred from two mitochondrial genes. Southeast. Naturalis. 2012; 11:297–310. https://doi.org/10.1656/058.011.0211.

38. Du WG, Hu LJ, Lu JL, Zhu LJ. Effects of incubation temperature on embryonic development rate, sex ratio and post-hatching growth in the Chinese three-keeled pond turtle, *Chinemys reevesii*. Aquaculture. 2007; 272:747–753. https://doi.org/10.1016/j.aquaculture.2007.09.009.

39. Schmaltz G, Somers CM, Sharma P, Quinn JS. Non-destructive sampling of maternal DNA from the external shell of bird eggs. Conservation Genetics. 2006; 7:543–549. https://doi.org/10.1007/s10592-005-9065-x.

40. Gagneux P, Boesch C, Woodruff D. Microsatellite scoring errors associated with non-invasive genotyping based on nuclear DNA amplified from shed hair. Molecular Ecology. 1997; 6:861–868. https://doi.org/10.1111/j.1365-294X.1997.tb00140.x.

41. Goossens B, Waits L, Taberlet P. Plucked hair samples as a source of DNA: reliability of dinucleotide microsatellite genotyping. Molecular Ecology. 1998; 7:1237–1241. https://doi.org/10.1046/j.1365-294x.1998.00407.x

42. Egloff C, Labrosse A, Hebert C, Crump D. A nondestructive method for obtaining maternal DNA from avian eggshells and its application to embryonic viability determination in hering gulls (*Larus argentatus*). Molecular Ecology Resources. 2009; 9: 19–27. https://doi.org/10.1111/j.1755-0998.2008.02214.x.

43. Shiau T, Hou P, Wu S, Tu M. A survey on alien pet reptiles in Taiwan. Taiwania. 2006; 51: 71–80.

44. Langton TES, Atkins W, Herbert C. On the distribution, ecology and management of non-native reptiles and amphibians in the London Area. Part 1. Distribution and predator/prey impacts. London Naturalist. 2011; 90:83–156.

45. Kopecký O, Kalous L, Patoka J. Establishment risk from pet-trade freshwater turtles in the European Union. Knowledge and Management of Aquatic Ecosystems. 2013; 410:1–11. https://doi.org/10.1051/kmae/2013057.

46. Iftime A, Iftime O. Alien fish, amphibian and reptile species in Romania and their invasive status: a review with new data. Travaux du Muséum National d'Histoire Naturelle "Grigore Antipa". 2021; 64:131–186. https://doi.org/10.3897/travaux.64.e67558.

47. Global Biodiversity Information Facility (GBIF). 2022 [cited 2022 February 04]. https://www.gbif.org.

48. Ernst CH, Lovich JE. Turtles of the united states and Canada. Johns Hopkins University Press. Baltimore, USA. 2009; pp. 387–392.

49. Munscher E, Walde AD, Stratmann T, Butterfield B. Exceptional growth rates observed in immature *Pseudemys* from a protected spring system in Florida. Herpetology Notes. 2015; 8:133–140.

50. Cadi A, Joly P. Impact of the introduction of the red-eared slider (*Trachemys scripta elegans*) on survival rates of the European pond turtle (*Emys orbicularis*). Biodiversity & Conservation. 2003; 13:2511–2518. https://doi.org/10.1023/B:BIOC.0000048451.07820.9c.

51. Silbernagel C, Clifford DL, Bettaso J, Worth S, Foley J. Prevalence of selected pathogens in western pond turtles and sympatric introduced red-eared sliders in California, USA. Diseases of aquatic organisms. 2013; 107:37–47. https://doi.org/10.3354/dao02663.

52. Jo SI, Na S, An CK, Kim HJ, Jeong YJ, Lim YM, et al. Comparison analysis for using the habitat pattern between the Korean endangered species, *Mauremys reevesii*, and the exotic species, *Trachemys scripta elegans*. Korean Journal of Environment and Ecology. 2017; 31(4):397–408. https://doi.org/10.13047/KJEE.2017.31.4.397.

53. Wimalasena SHMP, Shin GW, Hossain S, Heo GJ. Potential enterotoxicity and antimicrobial resistance pattern of Aeromonas species isolated from pet turtles and their

environment. Journal of Veterinary Medical Science. 2017; 79:921–926. https://doi.org/10.1292/jvms.16-0493.

Figure 1

Click here to access/download;Figure;Fig 1.jpg

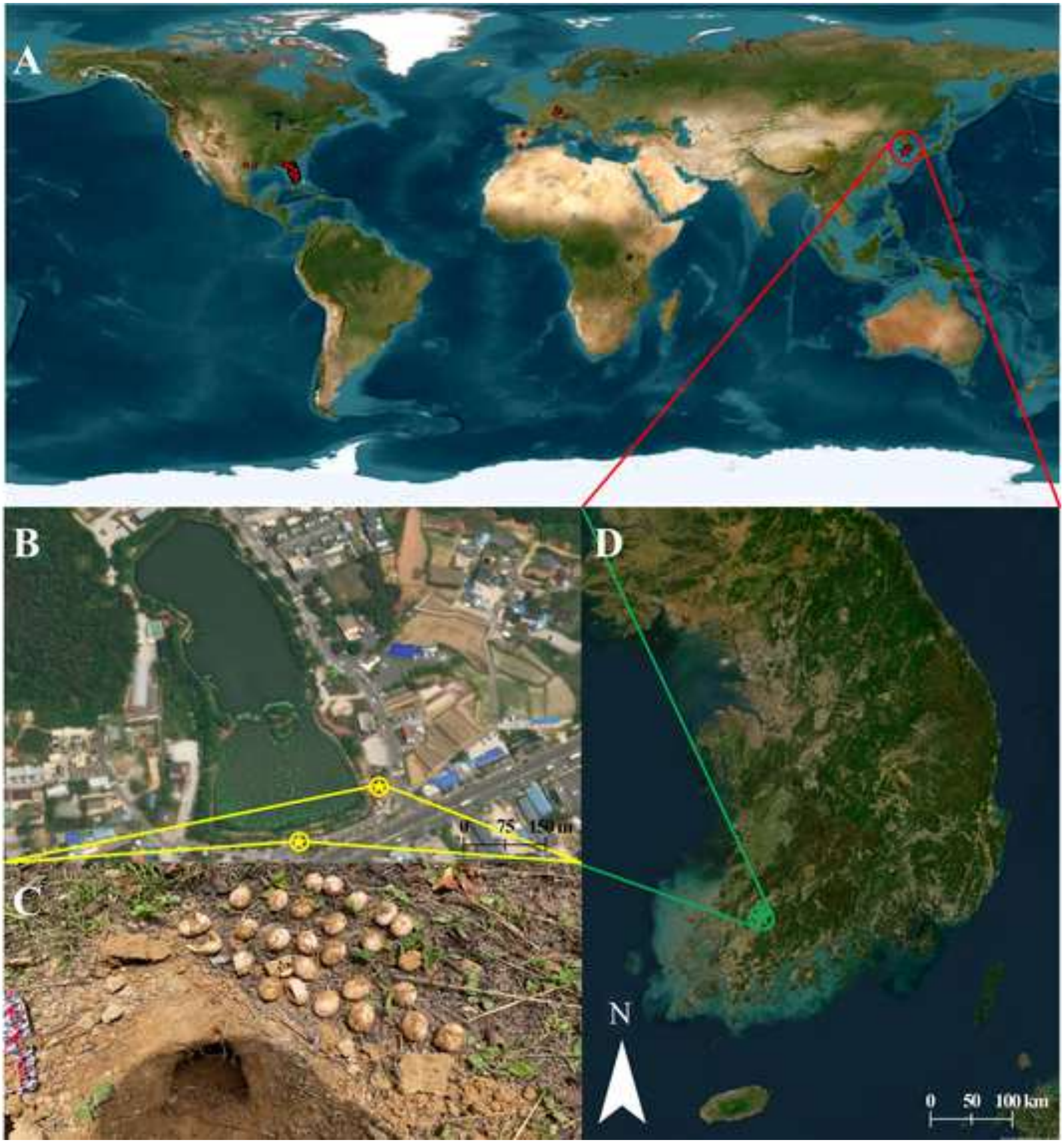

Figure 2 Click here to access/download;Figure;Fig 2.jpg

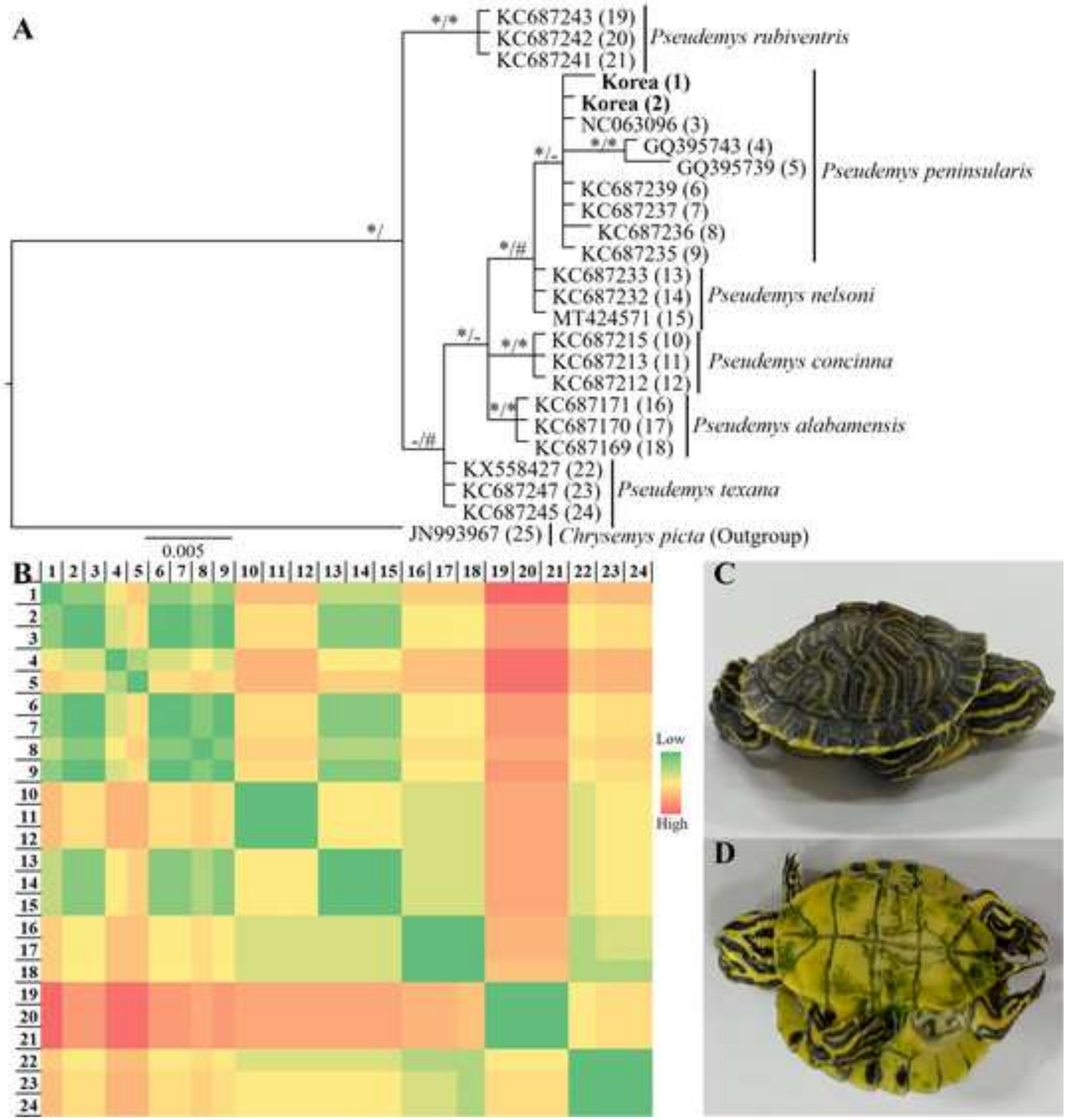

Figure 3

Click here to access/download;Figure;Fig 3.jpg

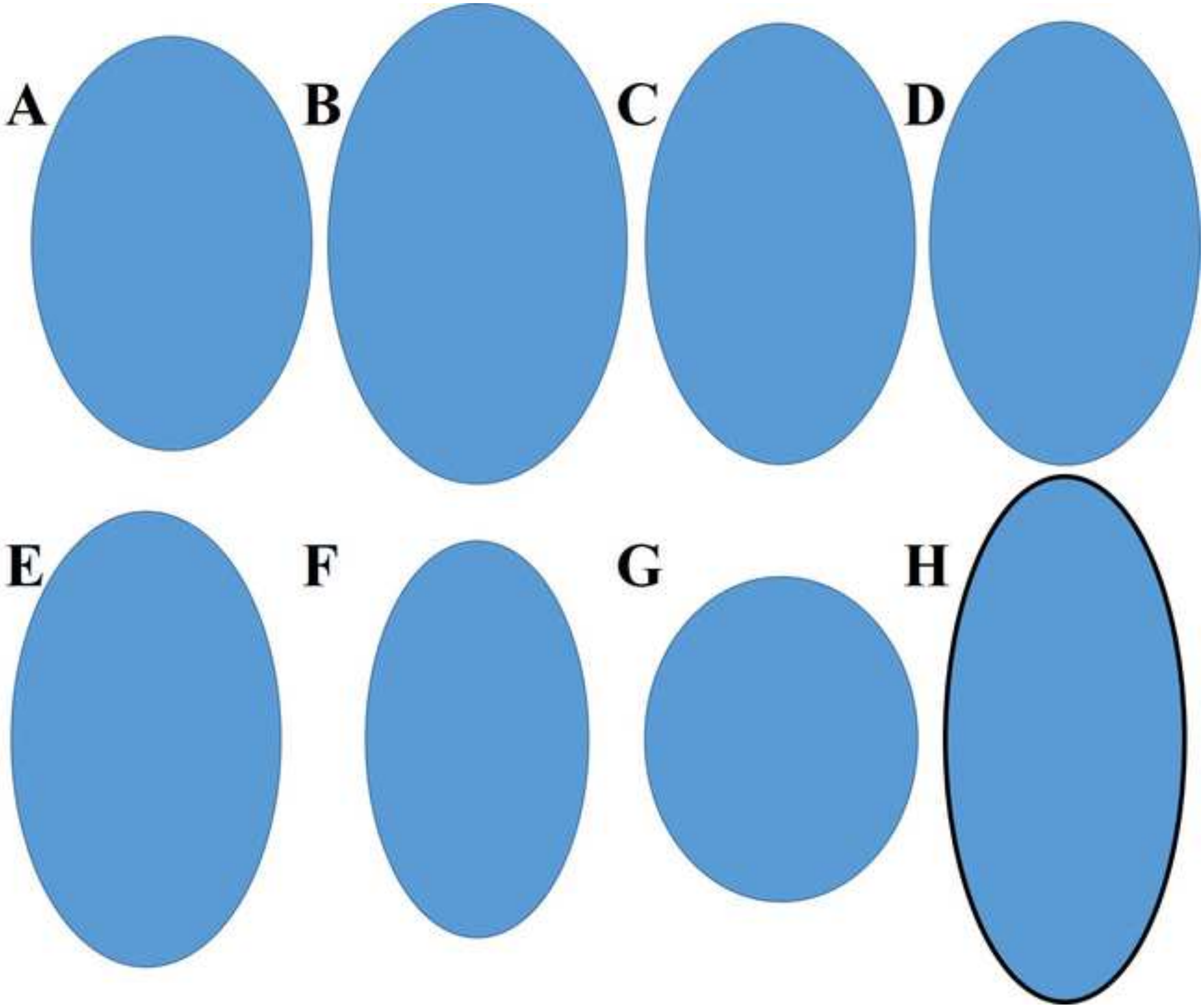

Supplement: Attachment — Submitted filename: PONE-D-22-32805_reviewer.pdf [file pone.0281808.s001.pdf]
