# Supplementary material for: Confirmation of the local establishment of alien invasive turtle, Pseudemys peninsularis, in South Korea, using eggshell DNA
Source: PLoS One. 2023 Feb 16;18(2):e0281808. doi: 10.1371/journal.pone.0281808 (PMC9934327; doi:10.1371/journal.pone.0281808)
Supplement: Attachment — Submitted filename: Response to editor and reviewers comments_MMR.doc [file pone.0281808.s002.doc]

**Point-by-point response to academic editor and reviewer’s comments**

**Response to comments from the academic editor**

**Comment 1:** Please ensure that your manuscript meets PLOS ONE's style requirements, including those for file naming. The PLOS ONE style templates can be found at 
<https://journals.plos.org/plosone/s/file?id=wjVg/PLOSOne_formatting_sample_main_body.pdf> and 
<https://journals.plos.org/plosone/s/file?id=ba62/PLOSOne_formatting_sample_title_authors_affiliations.pdf>

***Response*:** Thanks for your comment. We have revised the manuscript accordingly. We have modified the symbols of the author’s affiliation, font sizes of headings and subheadings, spacing of the first lines of all paragraphs, formatted tables, etc., following the guidelines.

**Comment 2:** Thank you for stating the following in the Acknowledgments Section of your manuscript: 
"This work was supported by Korea Environment Industry & Technology Institute (KEITI) through the Project for the Development of Biological Diversity Threats Outbreak Management Technology (RE201807039), funded by the Korea Ministry of Environment (MOE). "

We note that you have provided funding information that is not currently declared in your Funding Statement. However, funding information should not appear in the Acknowledgments section or other areas of your manuscript. We will only publish funding information present in the Funding Statement section of the online submission form. 
Please remove any funding-related text from the manuscript and let us know how you would like to update your Funding Statement. Currently, your Funding Statement reads as follows: 
"H-CS: RE201807039; Korea Environment Industry & Technology Institute. The funders had no role in study design, data collection and analysis, decision to publish, or preparation of the manuscript."

Please include your amended statements within your cover letter; we will change the online submission form on your behalf.

***Response*:** We appreciate your suggestion. We have deleted the ‘Acknowledgements’ section and omitted funding information in the revised manuscript. We have included the amended 'Funding Statement' in the cover letter.

**Comment 3:** In your Data Availability statement, you have not specified where the minimal data set underlying the results described in your manuscript can be found. PLOS defines a study's minimal data set as the underlying data used to reach the conclusions drawn in the manuscript and any additional data required to replicate the reported study findings in their entirety. All PLOS journals require that the minimal data set be made fully available. For more information about our data policy, please see <http://journals.plos.org/plosone/s/data-availability>.

"Upon re-submitting your revised manuscript, please upload your study’s minimal underlying data set as either Supporting Information files or to a stable, public repository and include the relevant URLs, DOIs, or accession numbers within your revised cover letter. For a list of acceptable repositories, please see [http://journals.plos.org/plosone/s/data-availability#loc-recommended-repositories](http://journals.plos.org/plosone/s/data-availability#loc-recommended-repositories). Any potentially identifying patient information must be fully anonymized.

Important: If there are ethical or legal restrictions to sharing your data publicly, please explain these restrictions in detail. Please see our guidelines for more information on what we consider unacceptable restrictions to publicly sharing data: [http://journals.plos.org/plosone/s/data-availability#loc-unacceptable-data-access-restrictions](http://journals.plos.org/plosone/s/data-availability#loc-unacceptable-data-access-restrictions). Note that it is not acceptable for the authors to be the sole named individuals responsible for ensuring data access.

We will update your Data Availability statement to reflect the information you provide in your cover letter.

***Response*:** We highly appreciate your comment. We have modified the ‘Data Availability Statement’ accordingly. All relevant data are within the manuscript. Please, check line 287.

**Comment 4:** We note that Figures 1a,1b and 1d in your submission contain [map/satellite] images which may be copyrighted. All PLOS content is published under the Creative Commons Attribution License (CC BY 4.0), which means that the manuscript, images, and Supporting Information files will be freely available online, and any third party is permitted to access, download, copy, distribute, and use these materials in any way, even commercially, with proper attribution. For these reasons, we cannot publish previously copyrighted maps or satellite images created using proprietary data, such as Google software (Google Maps, Street View, and Earth). For more information, see our copyright guidelines: <http://journals.plos.org/plosone/s/licenses-and-copyright>.
We require you to either (1) present written permission from the copyright holder to publish these figures specifically under the CC BY 4.0 license, or (2) remove the figures from your submission:
a. You may seek permission from the original copyright holder of Figures 1a,1b and 1d to publish the content specifically under the CC BY 4.0 license.  
We recommend that you contact the original copyright holder with the Content Permission Form (<http://journals.plos.org/plosone/s/file?id=7c09/content-permission-form.pdf>) and the following text:
“I request permission for the open-access journal PLOS ONE to publish XXX under the Creative Commons Attribution License (CCAL) CC BY 4.0 (<http://creativecommons.org/licenses/by/4.0/>). Please be aware that this license allows unrestricted use and distribution, even commercially, by third parties. Please reply and provide explicit written permission to publish XXX under a CC BY license and complete the attached form.”
Please upload the completed Content Permission Form or other proof of granted permissions as an "Other" file with your submission.
In the figure caption of the copyrighted figure, please include the following text: “Reprinted from [ref] under a CC BY license, with permission from [name of publisher], original copyright [original copyright year].”
b. If you are unable to obtain permission from the original copyright holder to publish these figures under the CC BY 4.0 license or if the copyright holder’s requirements are incompatible with the CC BY 4.0 license, please either i) remove the figure or ii) supply a replacement figure that complies with the CC BY 4.0 license. Please check copyright information on all replacement figures and update the figure caption with source information. If applicable, please specify in the figure caption text when a figure is similar but not identical to the original image and is therefore for illustrative purposes only.
The following resources for replacing copyrighted map figures may be helpful:
USGS National Map Viewer (public domain): <http://viewer.nationalmap.gov/viewer/>
The Gateway to Astronaut Photography of Earth (public domain): <http://eol.jsc.nasa.gov/sseop/clickmap/>
Maps at the CIA (public domain): <https://www.cia.gov/library/publications/the-world-factbook/index.html> and <https://www.cia.gov/library/publications/cia-maps-publications/index.html>
NASA Earth Observatory (public domain): <http://earthobservatory.nasa.gov/>
Landsat: <http://landsat.visibleearth.nasa.gov/>
USGS EROS (Earth Resources Observatory and Science (EROS) Center) (public domain): [http://eros.usgs.gov/#](http://eros.usgs.gov/)
Natural Earth (public domain): <http://www.naturalearthdata.com/>

***Response*:** We appreciate your concern. The maps and satellite images were generated using Arcmap 10.8.1 (ESRI, USA; <https://support.esri.com/en/products/desktop/arcgis-desktop/arcmap/10-8-1>), which is free to use and we referred the source in the figure caption. Please, check lines 108 – 113 of the revised manuscript.

**Comment 5:** We note that you have referenced Unpublished in page 6 which has currently not yet been accepted for publication. Please remove this from your References and amend this to state in the body of your manuscript: (ie “Bewick et al. [Unpublished]”) as detailed online in our guide for authors
[http://journals.plos.org/plosone/s/submission-guidelines#loc-reference-style](http://journals.plos.org/plosone/s/submission-guidelines#loc-reference-style)

***Response*:** Thanks for your advice. We have changed the reference following your suggestion. Please, check Table 1 of the revised manuscript.

**Comment 6:** Please review your reference list to ensure that it is complete and correct. If you have cited papers that have been retracted, please include the rationale for doing so in the manuscript text, or remove these references and replace them with relevant current references. Any changes to the reference list should be mentioned in the rebuttal letter that accompanies your revised manuscript. If you need to cite a retracted article, indicate the article’s retracted status in the References list and also include a citation and full reference for the retraction notice.

***Response*:** We appreciate your suggestion. We have checked the references and confirmed their complete and correctness. Besides, we have added one additional reference suggested by the reviewer. Please, check lines 314 – 316. We have mentioned that in the response of reviewer’s comments too.

**Response to comments from the Reviewer:**

**Comment 1:** I read the MS titled "Confirmation of the local establishment of alien invasive turtle, *Pseudemys peninsularis*, in South Korea using eggshell DNA" with interest. Congratulations on a catchy article. I see the merit of the given study for further monitoring and research activities and it is obvious that the goal of the paper should be overlapped to future practice widely. The text is clearly written, and the analyses and outputs are well-presented and described. Just a few mine minor comments are highlighted directly in the PDF copy attached. Check the format of the listed references carefully. The comments and suggestions are not plenty and easy to follow. After incorporating the suggestions in the text, it is my great pleasure to recommend this MS for publication in PLOS ONE.

***Response*:** We highly appreciate your reviewing efforts and positive comments. We have modified the manuscript as per your instructions and tried to resolve all issues you mentioned. Herein, we are responding to each of your concerns point by point.

**Comment 2:** ‘Not only, un wanted and undesired pets are also released’

***Response*:** Thanks for your comment. We have added the information in our revised manuscript. Please, check line 27 – 28.

**Comment 3:** ‘I suggest to add a brief note on the focused legislative regulations which are unfortunately ineffective in many cases. See: Patoka, J., Magalhães, A. L. B., Kouba, A., Faulkes, Z., Jerikho, R., & Vitule, J. R. S. (2018). Invasive aquatic pets: failed policies increase risks of harmful invasions. Biodiversity and Conservation, 27(11), 3037-3046.’

***Response*:** We highly appreciate your advice. We have added the suggested brief note in the updated manuscript. Please, check lines 50 – 54.

**Comment 4:** ‘Do you know how many turtle species are pet-trade in the country?’

***Response*:** Thanks for your question. There are 110 turtle species are pet-trade in the country. We also have included the information in the manuscript. Please, check line 57 of the revised manuscript.

**Comment 5:** ‘This info has to be moved to the results section.’

***Response*:** We appreciate your suggestion. We have transferred it to the results section as per your suggestion. Please, check line 194 of the manuscript.

**Comment 6:** ‘Since the captions have to be self-explaining, the full name is required in each caption where is the first mentioned.’

***Response*:** Thanks for your valuable comment. We have modified the scientific name accordingly. Please, check lines 155 – 156.

**Comment 7:** ‘Full name in this cell’

***Response*:** Thanks for your advice. We have modified it accordingly. Please, check Table 1.

**Comment 8:** ‘Unpublished’

***Response*:** We highly appreciate your comment. We have corrected the word accordingly. Please, check Table 1.

**Comment 9:** ‘The full name here’

***Response*:** Thanks for the suggestion. We have modified the scientific name. Please, check line 190 – 191 of the revised manuscript.

**Comment 10:** ‘Italicized’

***Response*:** Thanks for the advice. We have modified the scientific name. Please, check line 191 of the revised manuscript.

**Comment 11:** ‘Italicized’

***Response*:** Thanks for the suggestion. We have modified the scientific name. Please, check line 205 of the revised manuscript.

**Comment 12:** ‘abbreviated *Graptemys*’

***Response*:** We appreciate your suggestion. We have abbreviated the scientific name. Please, check line 206 of the revised manuscript.

**Comment 13:** ‘Here, *P. concinna* is adequate’

***Response*:** Thanks for the advice. We have modified the scientific name. Please, check line 211 of the revised manuscript.

**Comment 14:** ‘abbreviated Graptemys’

***Response*:** Thanks for the suggestion. We have modified the scientific name. Please, check line 211 – 212 of the revised manuscript.

**Comment 15:** ‘Italicized’

***Response*:** We appreciate your advice. We have italicized the scientific name. Please, check line 254 of the revised manuscript.

**Comment 16:** ‘Check all these species if cited above or not. If yes, use the genus abbreviation.’

***Response*:** Thanks for the advice. We have checked and modified the scientific names accordingly. Please, check lines 257 – 258 of the revised manuscript.

**Comment 17:** ‘Change i to y.’

***Response*:** Thanks for the comment. We have rechecked the whole manuscript and corrected the scientific names if needed. Herein, we have abbreviated the genus name following your previous suggestion. Please, check line 257 of the revised manuscript.

**Comment 18:** ‘including alien and also invasive species.’

***Response*:** We appreciate your suggestion. We have modified the sentence accordingly. Please, check lines 280 – 281 of the revised manuscript.

**Comment 19:** ‘Change m to M.’

***Response*:** We highly appreciate your comment. We have modified the name accordingly. Please, check line 292 of the manuscript.

**Comment 20:** ‘Change y to Y.’

***Response*:** Thanks for the advice. We have corrected it accordingly. Please, check line 302of the revised manuscript.

**Comment 21:** ‘Italicized.’

***Response*:** We appreciate your suggestion. We have corrected the scientific name and turned it into italicized. Please, check line 308 of the revised manuscript.

**Comment 22:** ‘Family, thus regular and not italicized.’

***Response*:** Thanks for the comment. We have turned it in to non-italicized regular font. Please, check line 308 of the revised manuscript.

**Comment 23:** ‘Just article No. 12:13143.’

***Response*:** We appreciate your comment. We have corrected accordingly. Please, check line 315 of the revised manuscript.

**Comment 24:** ‘- or –?’

***Response*:** We highly appreciate your comment. We have corrected accordingly. Please, check line 319 of the revised manuscript.

**Comment 25:** ‘dtto.’

***Response*:** Thanks for your comment. We have corrected accordingly. Please, check line 327 of the revised manuscript.

**Comment 26:** ‘Change c to C and b to B.’

***Response*:** We appreciate your advice. We have corrected accordingly. Please, check line 349 of the revised manuscript.

**Comment 27:** ‘Why abbreviated.’

***Response*:** We highly appreciate your comment. We have corrected accordingly. Please, check lines 410 – 411 of the revised manuscript.

**Comment 28:** ‘Just article No. 410:2.’

***Response*:** Thanks for your suggestion. We have corrected accordingly. Please, check line 435 of the revised manuscript.

**Comment 29:** ‘Aquatic Organisms.’

***Response*:** We appreciate your advice. We have corrected accordingly. Please, check line 453 of the revised manuscript.

**Comment 30:** ‘Italicized.’

***Response*:** We appreciate your comment. We have corrected accordingly. Please, check line 460 of the revised manuscript.
